# NEIGHBORHOOD CONTEXT AND LATER-LIFE WELL-BEING: OBJECTIVE AND SUBJECTIVE INDICATORS

**DOI:** 10.1093/geroni/igz038.244

**Published:** 2019-11-08

**Authors:** Noah J Webster, Christine Mair, Malcolm Cutchin

**Affiliations:** 1 University of Michigan, Ann Arbor, Michigan, United States; 2 University of Maryland, Baltimore County, Baltimore, Maryland, United States; 3 Wayne State University, Detroit, Michigan, United States

## Abstract

Neighborhoods are known to shape well-being across the life course, particularly in later life. Yet, neighborhoods remain an underutilized focus in public health interventions due to lack of measurement specificity and understanding of mechanisms across well-being outcomes. This symposium brings together four complementary papers from social work, sociology, and psychology that incorporate multiple (objective and/or subjective) indicators of neighborhood context from national to locally-based samples to predict diverse forms of well-being. Lehning and colleagues use objective (Census) indicators of neighborhoods from the National Neighborhood Change Database, combined with data from the National Health and Aging Trends Study (N=7,197), to explore links between changing neighborhood age composition and older adults’ self-rated health.. Sharifian and colleagues examine the associations between perceived neighborhood characteristics (social cohesion, physical disorder) and cognition directly and indirectly through mental health pathways (depressive symptoms, anxiety) using the Health and Retirement Study. Webster and colleagues examine links between objective (Census) and subjective (perceived safety) neighborhood indicators and social well-being using data from the Detroit-based Social Relations Study (N=259). Mair et al. combine objective neighborhood-level data with individual-level data from the Healthy Aging in Neighborhoods of Diversity across the Life Span dataset to examine direct and indirect effects of neighborhood context on obesity risk among N=2,707 middle-age and older Baltimore City residents. These papers will be discussed by Malcolm Cutchin, a health geographer and social gerontologist, who will provide an interdisciplinary reflection of neighborhood measurement and use neighborhoods as a platform to promote diverse forms of well-being in later life.

